# Draft Genome Sequence of Lactobacillus kefiri SGL 13, a Potential Probiotic Strain Isolated from Kefir Grains

**DOI:** 10.1128/MRA.00937-18

**Published:** 2018-08-02

**Authors:** Federica Federici, Laura Manna, Eleonora Rizzi, Elena Galantini, Umberto Marini

**Affiliations:** a Sintal Dietetics s.r.l., Castelnuovo Vomano, Teramo, Italy; Queens College

## Abstract

In this report, we present the draft genome sequence of a newly discovered potential probiotic strain of Lactobacillus kefiri, SGL 13, isolated from kefir grains. Antibiotic resistance analysis did not reveal evidence of interspecific horizontal gene transfer since the identified bacitracin resistance gene does not have mobile genetic elements.

## ANNOUNCEMENT

Kefir grains are composed of a complex community of microorganisms confined in a matrix of polysaccharides and proteins (1, 2). The drink obtained by fermentation of milk using these grains is called “kefir,” and its consumption has been associated with several health-promoting properties (3, 4), such as immunological, antimicrobial, antitumoral, and hypocholesterolemic effects.

Lactobacillus kefiri SGL 13 was isolated from kefir grains with the aim to study its beneficial properties since it is the most predominant species in kefir-fermented milk (5).

To our knowledge, this is the first genome sequence published for this species, even though L. kefiri constitutes a species of great interest for the development of new probiotic agents.

The strain SGL 13 was cultivated in de Man-Rogosa-Sharpe (MRS) broth at 30°C for 24 hours before biobanking and DNA extraction. Genomic DNA was purified with a Wizard genomic DNA purification kit (Promega) according to the manufacturer’s instructions. For genome sequencing, a paired-end library (Nextera XT) was sequenced with a HiSeq 2500 (Illumina) system to provide high-fidelity short reads. This sequencing approach was coupled with sequencing of a 6-kb genomic library in one single-molecule real-time (SMRT) cell of a PacBio RS II platform (Pacific Biosciences), leading to long reads used to assemble scaffolds. Illumina reads were filtered for quality standards using CASAVA 1.8.3. PacBio reads were filtered with BBMap 34.46 and assembled using ABySS 1.5.1 to create contigs, for which Illumina reads were aligned using BLASR (6) to make superscaffolds for which order and orientation were checked with SSPACE-LongRead scaffolder 1.0 (7, 8). Missing regions in the superscaffolds were filled using GapFiller 1.10 software (9). The genome was assembled into 10 final scaffolds, a total of 2,570,721 bp. This value is in line with the genome size of the type strain of the species, L. kefiri DSM 20587 (JCM 5818), the only other strain for which the genome sequence is available. Indeed, two independent sequencing projects are available for the same strain, GenBank BioProject no. PRJNA222257 for L. kefiri DSM 20587 (2,299,330 bp) and GenBank BioProject no. PRJDB803 for L. kefiri JCM 5818 (2,501,980 bp).

The SGL 13 genome was annotated using Prokka software (10) with default parameters, and the outputs were obtained in GenBank format. The L. kefiri SGL 13 draft genome consists of 10 scaffolds, with a coverage of 377× and a G+C content of 41.9%. The largest scaffold obtained is 2,408,480 bp. The total L. kefiri SGL 13 draft genome contains 2,506 coding sequences, of which 1,609 have a known function. Comparison of SGL 13 with L. kefiri DSM 20587, whose genome is composed of 95 scaffolds and thus is quite fractionated, revealed the presence of plasmids, but more investigations will be necessary to determine their number and structure.

Antibiotic resistance characterization was performed using BLAST (11) software against the ARDB database (12) and showed a putative resistance to bacitracin (undecaprenyl pyrophosphate phosphatases [UppPs]). The gene has no elements potentially responsible for interspecific horizontal gene transfer in syntenic regions (±500 bp from the start and stop codons) (13). The annotated shotgun sequence was exported in GenBank format and deposited on the NCBI whole-genome shotgun (WGS) portal.

### Data availability.

This whole-genome shotgun project has been deposited in the NCBI repository under the BioProject no. PRJNA467650.

## References

[1] Garrote GL, Abraham AG, De Antoni GL 2001 Chemical and microbiological characterisation of kefir grains. J Dairy Res 68:639–652. doi:10.1017/S0022029901005210. 11928960

[2] Garrote G, Abraham A, De Antoni G 2010 Microbial interactions in kefir: a natural probiotic drink. (no. 1980) *In* Mozzi F, Raya RR, Vignolo GM (ed), Biotechnology of lactic acid bacteria: novel applications. Wiley-Blackwell, Oxford, United Kingdom.

[3] Vinderola CG, Duarte J, Thangavel D, Perdigón G, Farnworth E, Matar C 2005 Immunomodulating capacity of kefir. J Dairy Res 72:195–202. doi:10.1017/S0022029905000828. 15909685

[4] Guzel-Seydim ZB, Kok-Tas T, Greene AK, Seydim AC 2011 Review: functional properties of kefir. Crit Rev Food Sci Nutr 51:261–268. doi:10.1080/10408390903579029. 21390946

[5] Garrote GL, Serradell MA, Abraham AG, Añon MC, Fossati CA, De Antoni GL 2005 Development of an immunochemical method to detect *Lactobacillus kefir*. Food Agric Immunol 16:221–233. doi:10.1080/09540100500244146.

[6] Chaisson MJ, Tesler G 2012 Mapping single molecule sequencing reads using basic local alignment with successive refinement (BLASR): application and theory. BMC Bioinformatics 13:238. doi:10.1186/1471-2105-13-238. 22988817 PMC3572422

[7] Boetzer M, Henkel CV, Jansen HJ, Butler D, Pirovano W 2011 Scaffolding pre-assembled contigs using SSPACE. Bioinformatics 27:578–579. doi:10.1093/bioinformatics/btq683. 21149342

[8] Boetzer M, Pirovano W 2014 SSPACE-LongRead: scaffolding bacterial draft genomes using long read sequence information. BMC Bioinformatics 15:211. doi:10.1186/1471-2105-15-211. 24950923 PMC4076250

[9] Boetzer M, Pirovano W 2012 Toward almost closed genomes with GapFiller. Genome Biol 13:R56. doi:10.1186/gb-2012-13-6-r56. 22731987 PMC3446322

[10] Seemann T 2014 Prokka: rapid prokaryotic genome annotation. Bioinformatics 30:2068–2069. doi:10.1093/bioinformatics/btu153. 24642063

[11] Altschul SF, Gish W, Miller W, Myers EW, Lipman DJ 1990 Basic local alignment search tool. J Mol Biol 215:403–410. doi:10.1016/S0022-2836(05)80360-2. 2231712

[12] Liu B, Pop M 2009 ARDB—Antibiotic Resistance Genes Database. Nucleic Acids Res 37:D443–D447. doi:10.1093/nar/gkn656. 18832362 PMC2686595

[13] Leplae R, Lima-Mendez G, Toussaint A 2010 ACLAME: a CLAssification of Mobile genetic Elements, update 2010. Nucleic Acids Res 38:D57–D61. doi:10.1093/nar/gkp938. 19933762 PMC2808911

